# Mosquito Cellular Factors and Functions in Mediating the Infectious entry of Chikungunya Virus

**DOI:** 10.1371/journal.pntd.0002050

**Published:** 2013-02-07

**Authors:** Regina Ching Hua Lee, Hapuarachchige Chanditha Hapuarachchi, Karen Caiyun Chen, Khairunnisa' Mohamed Hussain, Huixin Chen, Swee Ling Low, Lee Ching Ng, Raymond Lin, Mary Mah-Lee Ng, Justin Jang Hann Chu

**Affiliations:** 1 Laboratory of Molecular RNA Virology and Antiviral Strategies, Yong Loo Lin School of Medicine, National University Health System, National University of Singapore, Singapore, Singapore; 2 Enviromental Health Institute, National Environmental Agency, Singapore, Singapore; 3 National Public Health Laboratory, Ministry of Health, Singapore, Singapore; 4 Flavivirology Laboratory, Department of Microbiology, Yong Loo Lin School of Medicine, National University Health System, National University of Singapore, Singapore, Singapore; Centers for Disease Control and Prevention, United States of America

## Abstract

Chikungunya virus (CHIKV) is an arthropod-borne virus responsible for recent epidemics in the Asia Pacific regions. A customized gene expression microarray of 18,760 transcripts known to target *Aedes* mosquito genome was used to identify host genes that are differentially regulated during the infectious entry process of CHIKV infection on C6/36 mosquito cells. Several genes such as epsin I (EPN1), epidermal growth factor receptor pathway substrate 15 (EPS15) and Huntingtin interacting protein I (HIP1) were identified to be differentially expressed during CHIKV infection and known to be involved in clathrin-mediated endocytosis (CME). Transmission electron microscopy analyses further revealed the presence of CHIKV particles within invaginations of the plasma membrane, resembling clathrin-coated pits. Characterization of vesicles involved in the endocytic trafficking processes of CHIKV revealed the translocation of the virus particles to the early endosomes and subsequently to the late endosomes and lysosomes. Treatment with receptor-mediated endocytosis inhibitor, monodansylcadaverine and clathrin-associated drug inhibitors, chlorpromazine and dynasore inhibited CHIKV entry, whereas no inhibition was observed with caveolin-related drug inhibitors. Inhibition of CHIKV entry upon treatment with low-endosomal pH inhibitors indicated that low pH is essential for viral entry processes. CHIKV entry by clathrin-mediated endocytosis was validated via overexpression of a dominant-negative mutant of Eps15, in which infectious entry was reduced, while siRNA-based knockdown of genes associated with CME, low endosomal pH and RAB trafficking proteins exhibited significant levels of CHIKV inhibition. This study revealed, for the first time, that the infectious entry of CHIKV into mosquito cells is mediated by the clathrin-dependent endocytic pathway.

## Author Summary

Deciphering the much neglected aspects of cellular factors in contributing to the infectious entry of CHIKV into mosquito cells may enhance our understanding of the conservation or diversity of these host factors amongst mammalian and arthropod for successful CHIKV replication. The study revealed that the infectious entry of chikungunya virus (CHIKV) into mosquito cells is mediated by the clathrin-dependent endocytic pathway. A customized gene expression microarray known to target the *Aedes* mosquito genome was used to identify host genes that are differentially regulated upon CHIKV infection. A combination of bio-imaging studies and pharmacological inhibitors confirmed the involvement of clathrin-mediated endocytosis as well as the importance of low endosomal pH during CHIKV infectious entry. Furthermore, the clathrin heavy chain, Eps15, RAB5, RAB7 and vacuolar ATPase B are shown to be essential for the infectious entry process of CHIKV. This study aims to underline the importance of cellular factors, particularly those associated with clathrin-dependent endocytosis, in mediating the infectious entry of CHIKV into mosquito cells.

## Introduction

Chikungunya virus (CHIKV) is an arthropod-borne virus of the genus *Alphaviruses*, belonging to the family *Togaviridae*. It is an enveloped, single-stranded, positive-sense RNA virus with a genome size of approximately 12,000 nucleotides. CHIKV virions measure 60–70 nm in diameter and it contains a spherical capsid with icosahedral symmetry. The viral genome encodes for four non-structural (nsP1–P4) and five structural proteins (capsid, E1, E2, 6K and E3) [1], [2]. Embedded in the lipid bilayer surrounding the viral capsids, the E1 and E2 structural proteins enable the virus to be directed to host cells for attachment and fusion with cellular membranes during infectious entry processes [1], [2]. Chikungunya is defined as “bent walker” in Makonde, which refers to the hunched posture observed in patients suffering from persisting arthralgia [3], [4]. Symptoms typically develop from 4–7 days after the bite of an infected mosquito vector. Characterized by high fever, joint pain, headache, vomiting and maculopapular rash, acute CHIKV infection lasts approximately 1–10 days, while chronic CHIKV infection often results in polyarthralgia and myalgia that persist for long periods. Other CHIKV-associated complications reported include lymphopenia, severe skin lesions, lethal hepatitis and encephalitis, with severe neurological symptoms documented during recent outbreaks in Réunion Island [3], [4].

While human transmission of CHIKV occurs via *Aedes* (*Ae.*) mosquitoes, particularly *Ae. aegypti and Ae. albopictus*, other *Aedes* species such as *Ae. furcifer*, *Ae. taylori*, *Ae. luteocephalus*, *Ae. africanus* and *Ae. Neoafricanus* are involved in enzootic cycles [5], [6]. *Alphaviruses* can be broadly divided into the New World encephalitic viruses and Old World arthritogenic viruses [7], [8]. Along with other widely recognized Old World *alphaviruses* such as Sindbis (SINV), Semliki Forest (SFV), Ross River (RRV) viruses, CHIKV is responsible for high morbidity rates, accounting for millions of adverse, albeit non-fatal cases [3], [9], [10]. Genomic analysis of previously and recently identified clinical isolates revealed unique molecular features, most prominently a point mutation in the viral envelope E1 glycoprotein (E1-A226V) [9], which was suggested to increase the capability of viral fusion, assembly and tropism that aids in virus transmission [11], thus accounting for the selective advantage of the viral subtype. The presence of the A226V mutation in the CHIKV E1 gene was also reported during a major outbreak of CHIKV infection in the Indian state of Kerala [12]. Based on an SFV model of infection, replacement of the alanine residue at position 226 of the E1 envelope protein to valine was previously observed to affect membrane fusion and is believed to result in differential cholesterol dependence [10], [13].

Viruses can enter host cells through various pathways such as phagocytosis, macropinocytosis, and receptor-mediated endocytosis. Viruses have evolved the ability to penetrate and release the viral genome into the cell cytoplasm, after binding to the cellular receptor(s). Penetration for enveloped RNA viruses includes endocytosis and membrane fusion, the latter of which can either take place in a pH independent manner at the cell surface or within intracellular vesicles (pH-dependent). Majority of viruses require endocytic internalization for productive infection, with the virions being led to appropriate replication sites, thus bypassing many cytoplasmic barriers [14]. In particular, RNA viruses posses the ability to hijack multiple portals of cellular entry. Endocytic pathways such as clathrin-mediated, clathrin-independent, macropinocytosis, caveolar-mediated and caveolar-independent, have been shown to be utilized by numerous viruses [15], [16]. Other less characterized pathways also include lipid raft-mediated endocytosis, in which dynamin participation has been proposed but has not been determined [14].

Microarray studies performed on arboviruses and its mosquito vectors have been limited and aimed at enhancing diagnostics and understanding immune-based antiviral mechanisms [17], [18]. Such studies were previously performed to analyze gene expression profiles of mosquito midguts in response to Sindbis (SINV) infection, and genes associated with vesicle transport and immune cascades were observed to be involved during the infection [19].

Previous studies have been conducted to investigate the different entry pathways of *Alphaviruses* on various cell lines. SFV and Venezuelan equine encephalitis virus (VEEV) have been shown to enter mammalian cells through pH-dependent endocytic pathway [20]. Additionally, SINV was observed to infect both mammalian and mosquito cells at neutral pH [21], while VEEV was found to enter *Ae. albopictus* C710 mosquito cells via pH-dependent endocytosis [22]. Analyses of infectious CHIKV entry have been limited to mammalian cells, with several findings noting that CHIKV infection on HEK293T mammalian cells is independent of clathrin heavy chain and dependent of functional Eps15 [3], [4]. However, little is currently known about the infectious CHIKV entry process and pathway into mosquito cells. Deciphering the much neglected aspects of cellular factors in contributing to the infectious entry of CHIKV into mosquito cells may enhance our understanding on the conservation or diversity of these host factors amongst mammalian and arthropod cells for successful CHIKV replication.

This unprecedented study therefore aims to examine the infectious entry processes of CHIKV in mosquito cells. Different strategies targeting cellular endocytosis were used, including customized microarray profiling of mosquito genes involved in endocytic pathways, treatment with specific drug inhibitors, gene knockdown and expression of dominant negative cellular proteins. We demonstrated, for the first time, that CHIKV preferentially uses a clathrin-mediated and Eps15-dependent pathway to enter *Ae. albopictus* (C6/36) cells. We also showed the importance of endosomal pH acidification in CHIKV entry. Moreover, results from the siRNA-based knockdown of Rab5 and Rab7 genes suggested that CHIKV entry involves the trafficking of virus particles from early to late endosomes. The novelty of deciphering the infectious entry of CHIKV into C6/36 cells potentially allows for better understanding on the pathogenesis of CHIKV infection and the development of potential antiviral strategies.

## Materials and Methods

### Cell Culture

*Ae. albopictus* C6/36 cells (American Type Culture Collection) were maintained at 28°C in Leibovitz-15 (L-15) growth medium (Sigma-Aldrich Corp., St Louis, MO, USA) supplemented with 10% fetal bovine serum (FBS) (Hyclone, Cramlinton, UK). Baby hamster kidney (BHK-21) cells (American Type Culture Collection, ATCC CCL-10) were maintained at 37°C in Rosewell Park Memorial Institute (RPMI-) 1640 growth medium (Sigma-Aldrich Corp) supplemented with 10% FBS (Hyclone).The cells were passaged in T75 flasks (Nunc, Denmark) at a 1∶5 dilution every 3–4 days at 70–80% confluency. For experimental infections, C6/36 cells were seeded in T25 flasks to a confluency of 80% that achieved a cell density of ∼3×10^6^ cells/ml. The C6/36 cells were incubated at 37°C for 1.5 hours during virus infection, before being placed at 28°C throughout the remainder of the experiments, in line with the natural temperature for mosquitoes and mosquito cell incubation.

### Viruses and CHIKV Infection

Singapore/07/2008 CHIKV strain was obtained from National Public Health Laboratory, Ministry of Health, Singapore and propagated in C6/36 cells. Low passages of the virus were used throughout this study. CHIKV strains SGEHICHD122508 – (Accession No.: FJ445502.2) and SGEHIDSD67Y2008 – (Accession No.: EU441882.1) were obtained from Environmental Health Institute, National Environmental Agency, Singapore. These virus strains were propagated in C6/36 cells and utilized in low endosomal pH experiments. The virus titers were quantitated using viral infectious plaques assays performed on BHK-21 cells. Growth kinetics were performed on these three different CHIKV strains, with infected and mock-infected samples harvested at various time points of 0, 6, 12, 24, 36, 48, 72, 96 and 120 hours post infection (p.i) on C6/36 cells. A multiplicity of infection (MOI) of 10 was used for most of the experiments throughout the study, to allow for more accurate observations and better detection of CHIKV entry processes into host cells.

### Purification of CHIKV

Confluent monolayers of C6/36 cells were infected with CHIKV at an MOI of 10. At 24 hours p.i, the supernatant was harvested by centrifugation at 4,500 rcf for 10 mins. CHIKV particles were then concentrated and partially purified by using a centrifugal filter device (Millipore, Billerica, MA, USA) at 1,077 rcf for 2 hours. The partially purified viruses were then purified even further by sucrose gradient centrifugation at 74,766 rcf for 3 hours at 4°C. Finally, the purified virus pellet was resuspended in Tris buffer (50 mM Tris-HCl [pH 7.4]). The titer of the purified virus preparation was determined by viral infectious plaque assay on BHK-21 cells and was found to be 5×10^10^ PFU/ml. For negative staining of purified CHIKV preparation, 7.5×10^8^ PFU/15 µl of CHIKV was added to freshly glow discharged, carbon-coated grids, and stained with 2% uranyl acetate for 1 min. The grids were then air dried before viewing under the CM120 Biotwin transmission electron microscope (Philips).

### Infectious Virus Entry Assay and Drug Treatments

C6/36 cells growing on coverslips were incubated with CHIKV at an MOI of 10 for 1 hour at 4°C with gentle rocking. The cells were subsequently washed three times in ice-cold 1× phosphate buffer saline (PBS) to remove unbound viral particles, prior to further incubation for 1 hour at 37°C in growth medium to enable virus penetration. Extracellular virus particles that failed to enter into cells are then inactivated with acid glycine buffer (pH 2.8) (0.1 M potassium hydrogen phthalate and 0.1 M of HCl). Infectious virus entry was traced at different time points upon the addition of CHIKV to C6/36 cells for up to 1 hour post-infection and processed for either ultrastructural analysis via transmission electron microscopy or immunofluorescence assay.

C6/36 cells (1.2×10^6^) were seeded into 24-well plates, and incubated for 24 hours before the drug treatment assays were performed. Pre-treatment drug assays were performed in favour of co- and post-treatment studies, in order to ensure that potential CHIKV inhibition is most likely to occur at the entry step, as opposed to downstream infective phases, such as viral replication. Hence, to determine the effects of the drugs used to inhibit the CHIKV entry, C6/36 cells were pretreated with drugs at different concentrations for 3 hours at 37°C. The pharmacological inhibitors were then removed and cell monolayers were washed twice with 1× PBS, in order to eliminate the possibility of exposure of the virus to the inhibitors. This is to ensure minimal risk of the inhibitors directly influencing the viability of the virus and its subsequent entry into the cells. After 1.5 hours of virus infection at an MOI of 1, the cells were washed thrice with 1× PBS, replaced with fresh L-15 media and incubated for another 24 hours. At 24 hour p.i., supernatants from CHIKV-infected cells were harvested for viral infectious plaque assays. Three independent experiments were carried out for each set of drugs used. Inhibition of receptor- and/or clathrin- mediated endocytosis was performed through the use of chlorpromazine (42, 56, 70 & 84 µM) (Sigma Aldrich) [23], monodansylcadaverine (50, 100, 150 & 200 µM) (Sigma Aldrich) [24] and dynasore (5, 10, 50 & 100 µM) (Sigma Aldrich) [25]. Other inhibitors targeting alternative endocytic pathways included filipin (0.1, 0.5, 1.0, 1.5 & 2.0 µg/ml) (Sigma Aldrich) [26], nystatin (5, 10, 20 & 40 µM) (Sigma Aldrich) [26], methyl-β-cyclodextrin (2.5, 5.0, 7.5 & 10 µM) (Sigma Aldrich) [26] and EIPA (10, 25, 50 & 100 µM) (Sigma Aldrich) [27], [28]. CHIKV infected, 0.1% DMSO treated C6/36 cells acted as solvent control. Endosomal acidification was inhibited by drug treatment of C6/36 cells with concanamycin A (20, 60, 100, 150 & 300 nM –Singapore/07/2008 CHIKV strain) and (80, 100, 150 & 300 nM - CHIKV strains SGEHICHD122508 and SGEHIDSD67Y2008) (Sigma Aldrich) and bafilomycin A (0.1, 1.0, 2.0, 3.0 & 4.0 µM) (Sigma Aldrich) [29], [30]. Other inhibitors performed on C6/36 cells include colchicine (50, 100, 150 & 200 µM) (Sigma Aldrich) [31], nocodazole (1, 5, 10, 15 & 20 µM) (Sigma Aldrich) [32], cytochalasin B (0.1, 1.0, 1.5 & 2.0 µg/ml) (Sigma Aldrich) [23], cytochalasin D (1, 3, 5, 10 & 20 µg/ml) (Sigma Aldrich) [31] and nifedipine (40, 60, 80 & 100 µM) (Sigma Aldrich) [33].

### Microarray Gene Expression Analysis

Upon infection, C6/36 cells were harvested at 0 min, 15 mins, 30 mins and 120 mins post infection (pi). At 0 min pi, cells were harvested immediately upon virus inoculation. At each time point, C6/36 cells were washed with 2 ml of the pre-warmed (28°C) maintenance medium. After decanting the maintenance medium, 1 ml of Qiagen Cell Protect solution was added to each flask. Detached cells were transferred into a sterile 2 ml tube and were stored immediately at −80°C until total RNA extraction. Cells were homogenized in 350 µl RLT buffer in QIAshredder spin columns (Qiagen, Hilden, Germany) prior to total RNA extraction with Qiagen RNeasy Protect cell mini kit (Qiagen) according to manufacturer's instructions. Hundred nanograms of total RNA were used for probe synthesis of cy3-labeled cRNA, and hybridizations were carried out on an *Aedes* mosquito customized gene expression microarray (18760 transcripts from Vector Base *Aedes aegypti* database with 2 best probes per transcript) in Agilent GE 8×60K array format (Agilent Technologies, California, USA). Hybridization was carried out at 65°C for 17 hours in an Agilent hybridization oven at 10 rpm. After hybridization, microarrays chips were washed in gene expression wash buffer 1 for 1 min at room temperature and 1 min in gene expression wash buffer 2 at 37°C before scanning on the Agilent High Resolution Microarray Scanner (C-model). Raw signal data was extracted from the TIFF image with Agilent Feature Extraction Software (V10.7.1.1). The raw microarray data was processed and analyzed with Partek Genomics Suite (Partek, St Louis, Missouri, USA) to generate values representing fold changes in gene expression. An average of the duplicate values was used to calculate fold change, and each value was then assessed for its statistical significance, using analysis of variance (ANOVA). Host genes demonstrating at least a 1.5-fold change in expression upon CHIKV infection were selected for further investigation. Pathway analysis was subsequently detailed with Ingenuity Pathway Analysis (IPA) 9.0 (Ingenuity Systems 2011, Redwood City, California) and differentially regulated genes involved in the clathrin-mediated endocytic pathway were selected for pathway mapping.

### Transmission Electron Microscopy

To track the infectious entry process of CHIKV into C6/36 cells at various time points p.i, cells infected with CHIKV at an MOI of 10 were fixed with 2.5% glutaraldehyde (Agar Scientific, Stansted, UK) at 4°C for 20 mins, followed by scraping of the cells and subjecting them to longer fixation at 4°C overnight. The following day, cells were centrifuged and the pellet was washed with PBS and deionized water. The cell pellet was then post-fixed with 1% osmium tetroxide (Ted Pella, Redding, California, USA) and 1% potassium ferro-cyanide for 2 hours, followed by dehydration in an ascending graded series of ethanol and acetone, i.e. 25%, 50%, 75%, 95% and 100% for 10 mins at each concentration. On the following day, cells were infiltrated with resins by passing them through three changes of mixture, comprised of a combination of acetone, ethanol and araldite. The following day, cells were infiltrated with four changes of absolute embedding media with 1 hour incubation at room temperature, 40°C, 45°C and 50°C. After the last spin, cell pellet was resuspended in 100–200 µl of araldite. Mixture was embedded using the BEEN capsule (size 3) and was incubated at 60°C for 24 hours to allow polymerization. The samples were trimmed with an ultramicrotome (Reichert-Jung, New York, USA) and the sections were stained with 2% uranyl acetate and fixed with lead citrate. The stained sections were viewed under the Philip EM 208 transmission electron microscope and images were captured digitally with a dual view digital camera (Gatan, Werrendale, USA).

### Indirect Immunofluorescence Microscopy

For immunofluorescence microscopy, C6/36 cell monolayers were first grown on coverslips till 75% confluency. The cells were incubated at 4°C for 30 mins. The cells were allowed to bind to CHIKV at an MOI of 10 for 1 hour at 4°C to allow viral attachment to the cell surface before being shifted to 37°C for 10 mins to promote CHIKV entry into the cell. Cells were fixed in ice-cold methanol at 10 and 15 mins post entry of CHIKV. This is followed by three washes of cold PBS prior to immunofluorescence assay analyses. Rabbit polyclonal antibodies to clathrin (CLTC, Chemicon), early endosomal antigen 1 (EEA1; Novus Biologicals) and CHIKV E2 protein (customized CHIKV13893 B3 rabbit polyclonal, ProSci) were used for immunofluorescence assays. Texas Red (TR)- or FITC-conjugated secondary antibodies were used at a concentration of 1 µg/ml. Lysotracker, a dye for staining live cells were used at a concentration of 75 nM. The specimens were then viewed with Olympus IX81 motorized inverted epifluorescence microscope (Olympus, Tokyo, Japan) with an excitation wavelength of 543 nm for TR and 480 nm for FITC at 63× magnification.

### Cell Viability Assay

Cell viability upon drug treatments and siRNA transfection was assessed by the Cell Cytoxicity Assay – alamarBlue (Invitrogen, CA, USA) assay according to the manufacturer's recommendations. Briefly, C6/36 cells were seeded in 96-well cell culture plates and subsequently treated with individual siRNAs or drugs for 3 hours, before incubation with alamarBlue reagent solution for 2 hours at 37°C. After which, the plates were subjected to fluorescence detection, at an excitation wavelength of 540 nm–570 nm, and emission wavelength of 580 nm–610 nm (Tecan iControl Reader, Männedorf, Switzerland).

### Transfection of Plasmid DNA and siRNA into Cells

Plasmid constructs of dominant-negative Eps15 (GFP-EΔ95/295) was kindly provided by A. Benmerah, Pasteur Institute, and plasmid constructs backbone EGFP-C2 was purchased from Clontech (CA, USA). Transfections were performed by using Lipofectamine LTX reagents according to manufacturer's recommendation (Invitrogen). Briefly, C6/36 cells were grown on coverslips in 24-well tissue culture plates until 75% confluency. Then, 3.5 µg plasmid constructs were complexed with 4 µl Plus reagent in 25 µl OPTI-MEM medium (Gibco, New York, USA) for 15 mins at room temperature. The mixture was then added to 25 µl OPTI-MEM containing 2 µl Lipofectamine LTX (Invitrogen, USA). After incubation for another 15 mins, the DNA-liposome complexes were added to the cells, prior to further incubation for 3 hours at 37°C. One millilitre of complete growth medium was then added and incubated for another 24 hours before the virus entry assay was carried out.

Different siRNAs targeting various *Ae. albopictus* genes involved in endocytic processes were selected to perform reverse transfection assays in C6/36 cells, including CLTC (NCBI Accession: XM_001656826), RAB5 (NCBI Accession: XM_001658641), RAB7 (NCBI Accession: EF127648) and vacuolar ATPase B (NCBI Accession: AF092934). The siRNA gene sequences used in this study are, CLTC (CAAUAAAGAUAAUGCCCAU), RAB5 (CGAAUAUUGUGAUUGCGCU), RAB7 (CCUGGAGAAUAGGGCCGUA) and vacuolar ATPase B (GUCAUUCAAGGGAUAAUGU) (Sigma Aldrich). Reverse transfection assays on scrambled siRNA gene sequences were also performed simultaneously to confirm the specificity of the gene targeting siRNAs. The scrambled siRNA gene sequences used in this study are CLTC (ACAGAAUUAAACUACUUGC), RAB5 (ACAGUUUGAGGUACUGUUC), RAB7 (CUCAGAGGGUAACGUCGAG) and vacuolar ATPase B (CUGAAUAUCAGUGGUAUAG). Specific gene targeting siRNAs and scrambled siRNAs were dissolved in DEPC-treated reverse osmosis water to a final stock concentration of 100 µM, and incubated at room temperature for 30 mins with gentle agitation. Different siRNAs were diluted to desired working concentrations of 0.1 nM, 1 nM, 5 nM, 10 nM with serum-free media (Dharmacon, US) and transfection reagent (Dharmafect-1). The specific individual siRNAs that were directed against each of the respective genes were then transfected into C6/36 cells prior to being subjected to CHIKV infection after 48 hours post transfection. The supernatants were then harvested 24 hours p.i for plaque assays.

### RNA Quantification via qRT-PCR

Validation of gene expression was performed via qRT-PCR. Upon gene silencing, total RNA was extracted from C6/36 cells with RNeasy Extraction Kit (Qiagen). The samples were assayed in a 20 µl reaction mixture containing 10 µl SYBR Green Master Mix (Fermentas, US), 1 µl forward and reverse primer respectively, 1 µl RNA, 1 µl reverse transcriptase and 7 µl nuclease free water. A no-template control was also included. The cycling conditions for one-step SYBR Green-based RT-PCR consisted of a 30-min reverse transcription step at 44°C and 5 mins of Taq polymerase activation at 94°C, followed by 40 cycles of PCR with denaturation occurring at 94°C for 15 s and annealing and extension taking place at 60°C for 30 s. Following amplification, a melting curve analysis was performed to verify the melting temperature of PCR products amplified by the *Ae. albopictus* gene primer pairs. The primers pairs stated are CLTC (Forward, 5*′*-CGTTCGGCCAATGCTGC-3*′* , Reverse, 3*′*- GGGAAGTCGCTCTGCGCT-5*′* ), RAB5 (Forward, 5*′*-TCAGCGACAGGCATCGC-3*′* , Reverse, 3*′*-CAGCGGTTTTGGCCGAC-5*′* ), RAB7 (Forward, 5*′*-AACGAAGCGTGCCCAGCAGT-3*′* , Reverse, 3*′*-CCGGTTGTTGCGGTCTGCGT-5*′* ), vacuolar ATPase B (Forward, 5*′*-GCTCGGTCTTCGAGTCGCT-3*′* , Reverse, 3*′*-CAGTGTCAGGCGCGAGGTC-5*′* ) and actin controls (Forward, 5*′*-CCACCATGTACCCAGGAATC-3*′* , Reverse, 3*′*-CACCGATCCAGACGGAGTAT-5*′* ).

### Statistical Analysis

Where applicable, statistical analyses were performed on repeated measurements using the one-tailed Student's t-test. The significance level was set at p<0.05 (*), p<0.01 (**) or p<0.001 (***). Data shown throughout the study were obtained from three independent experiments.

## Results

### Microarray Gene Expression Analysis

**Figure 1:**
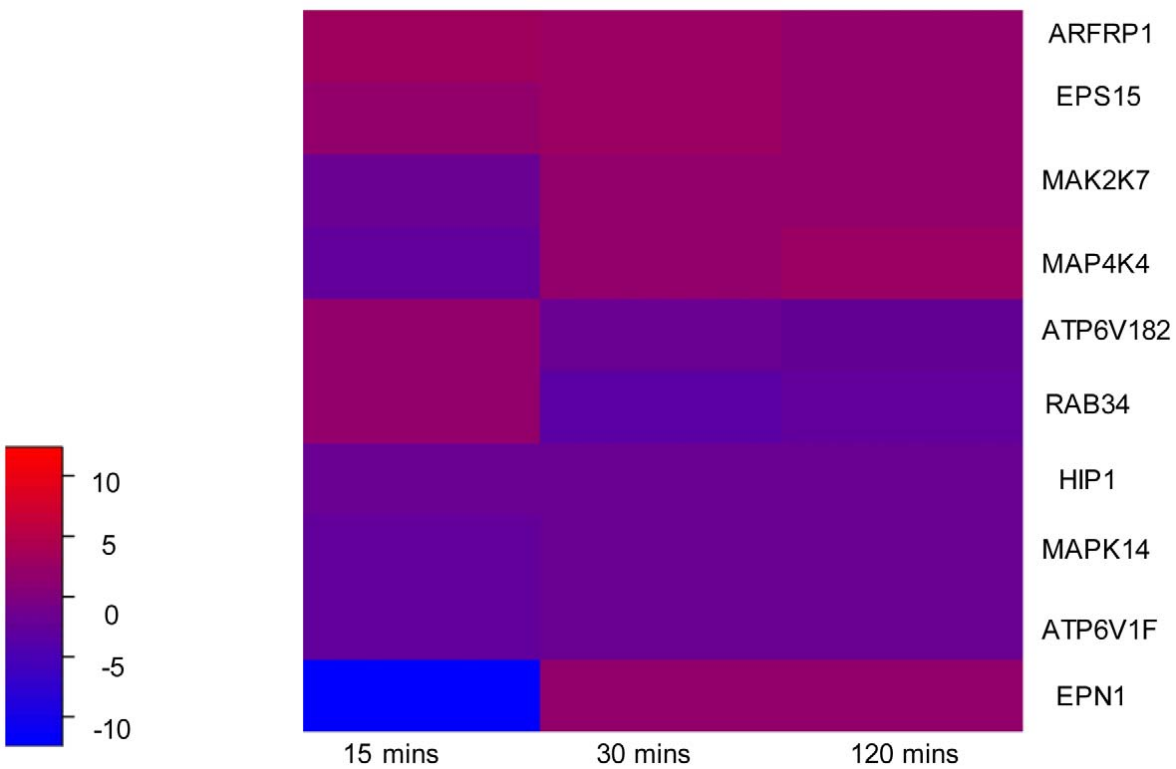
Heatmap displaying differential regulation of genes related to clathrin-mediated endocytotic pathway. Fold changes upon CHIKV infection, relative to mock-infected samples, are as depicted by the colour key: gene upregulation is denoted in red while gene downregulation is denoted in blue.

A customized gene expression microarray chip consisting of 18,760 transcripts targeting the *Ae. aegypti* mosquitoes was used to profile differentiated regulation levels of host genes necessary for the infectious entry of CHIKV. A total of 579 targeted mosquito genes were found to be differentially regulated – defined as fold change of less than −1.5 or more than 1.5 - upon CHIKV infection. Among these genes – many of which are known to be involved in generalized host immune responses, such as the IFN-associated pathway - are those related to clathrin-mediated endocytosis. Genes associated with other endocytic pathways, such as caveolin-mediated endocytosis and macropinocytosis were not observed to be differentially regulated based on the user-defined criteria. Standard housekeeping genes were also found to exhibit similar expression profiles upon CHIKV infection as mock-infected samples. A brief description of the reported mammalian-based functional roles and the fold changes upon various time points of CHIKV infection for each of the genes is shown in Table 1 and a heat map exhibiting the differential regulation of these genes across all time points of CHIKV infection is shown in Figure 1. These genes, or related genes, have also been mapped onto the clathrin-mediated endocytotic pathway, as shown in Figure S2. Genes known to be associated with clathrin-mediated endocytosis include epsin I (EPN1), epidermal growth factor receptor pathway substrate 15 (EPS15) and Huntingtin interacting protein I (HIP1). EPN1 and EPS15 were found to be upregulated while HIP1 was downregulated upon CHIKV infection. In addition, genes that targeted kinases (MAP2K7, MAP4K4 and MAPK14) were downregulated in the first 15 min of CHIKV infection, although MAP2K7 and MAP4K4 were subsequently found to be upregulated after 30 min and 120 min of infection. Genes involved in vesicle and endosomal transport, such as ATP6V1B2, ATP6V1F, ARFRP1 and RAB34 were also found to be differentially regulated during CHIKV infection. Taken together, analysis of the microarray data suggests the possible involvement of clathrin-mediated endocytosis in the infectious entry of CHIKV.

**Table 1:** Differentially expressed genes – associated with clathrin-mediated endocytosis - upon CHIKV infectious entry.

GENE SYMBOL	GENE NAME	GENE FUNCTIONS	CHIKV infection VS Mock-infected		
			15 mins p.i	30 mins p.i	120 mins p.i
ARFRP1	ADP-ribosylation factor related protein 1	A membrane-associated GTP-ase related to the ADP-ribosylation (ARF) and ARF-like (ARL genes).	3.169	2.827	1.924
ATPGV182	ATPASE, H+ transporting lysosomal 56/58 kDa, V1 subunit B2	A component of vacuolar ATPase (V-ATPase), a multisubunit enzyme that mediates acidification of eukaryotic intracellular organelles. V-ATPase dependent organelle acidification is necessary for such intracellular processes as protein sorting, zymogen activation, receptor-mediated endocytosis, and synaptic vesicle proton gradient generation.	1.621	−1.558	−2.303
ATP6V1F	ATPase, H+ transporting, lysosomal 14 kDA, V1 subunit F	Subunit of peripheral V1 complex of vacuolar ATPase essential for assembly or catalytic function. V-ATPases are compartments in eukaryotic cells.	−2.613	−1.723	−2.072
EPN1	Epsin 1	An endocytic accessory protein that interacts with the EPS15, the alpha subunit of the clathrin adaptor AP2 (AP2A1), and clathrin, as well as with other accessory proteins for the endocytosis of clathrin-coated vesicles. Binds to membranes enriched in phosphatidylinositol-4,5-bisphosphate [(Ptdins(4,5)P2)]. Modifies membrane curvature and facilitates the formation of clathrin-coated invaginations.	−12.375	1.665	1.876
EPS15	Epidermal growth factor receptor pathway substrate 15	Part of EGFR pathway, it is present at clathrin-coated pits and is involved in receptor-mediated endocytosis of EGF. It is involved in the internalization of ligand-inducible receptors of the receptor tyrosine kinase (RTK) type, in particular EGFR and plays a role in the assembly of clathrin-coated pits.	1.844	2.772	1.565
HIP1	Huntingtin interacting protein 1	Plays a role in clathrin-mediated endocytosis and trafficking and may play a functional role in the cell filament networks	−1.622	−1.622	−1.628
MAK2K7	Mitogen-activated protein kinase kinase 7	A dual specificity protein kinase that belongs to the MAP kinase kinase family, and is involved in the signal transduction mediating the cell responses to proinflammatory cytokines, and environmental stresses.	−1.798	1.733	1.784
MAP4K4	Mitogen-activated protein kinase kinase kinase kinase 4	A member of serine/threonine protein kinase family that may play a role in the response to environmental stress and cytokines such as TNF-alpha.	−2.807	1.674	2.477
MAPK14	Mitogen-activated protein kinase 14	A member of the MAP kinase family that responds to activation by environmental stress, pro-inflammatory cytokines and lipopolysaccharide (LPS) by phosphorylating a number of transcription factors, such as ELK1 and ATF2 and several downstream kinases, such as MAPKAPK2 and MAPKAPK5, and plays a critical role in the production of some cytokines, such as IL-6.	−2.580	−1.732	−1.999
RAB34	RAB34, member RAS oncogene family	RAB34 is a member of the RAB protein family, which are small GTPases that regulates vesicle budding, docking and fusion along endocytosis and exocytosis pathways.	1.779	−3.219	−2.611

The mammalian-based function(s) of the individual genes are reproduced from the Online Mendelian Inheritance in Man as of December 2011. The three right-most columns represent fold change values upon various timepoints of CHIKV infection, relative to mock-infected samples.

### Bio-imaging of CHIKV Entry Process

**Figure 2:**
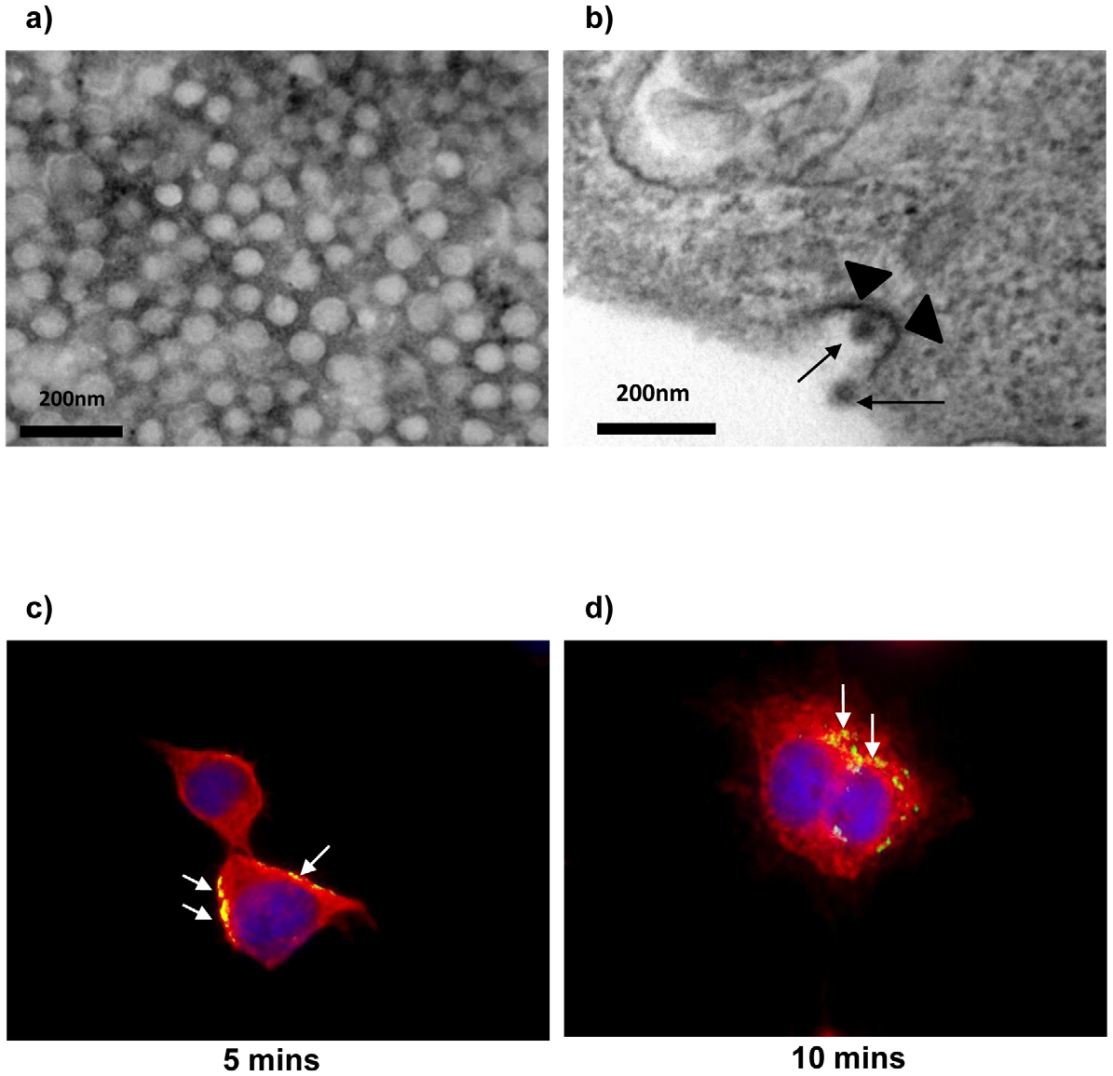
Bio-imaging analysis of CHIKV entry process into C6/36 cells using electron microscope (a and b) and immunofluorescene labeling. (a) CHIKV particles are negatively-stained and observed to be approximately 60–70 nm in size. (b) Attachment of CHIKV particles at the plasma membrane of C6/36 cells and uptake of CHIKV particles (arrow) by coated pits (arrowheads) (scale bar represent 200 nm). CHIKV viral particles (green) (c and d) are seen to co-localize with clathrin molecules (red) at 5 and 10 mins p.i. (arrows). Cell nuclei are stained with DAPI (blue).

Based on the microarray findings, we proceeded to employ a combination of bio-imaging techniques including transmission electron microscopy (TEM) and immunofluorescence assays, to further investigate the infectious entry processes of CHIKV. CHIKV was first prepared by a series of concentration and purification procedures. As revealed by negative staining of the virus preparation, a homogeneous population of CHIKV particles with a uniform size of 60–70 nm in diameter (Figure 2a) was obtained. The purified virus particles were subsequently used to map the infectious entry process of the virus into C6/36 cells. In order to visualize synchronized entry of CHIKV into cells, C6/36 cells were first incubated with CHIKV (MOI = 10) at 4°C for 1 hour. Low-temperature treatment allows binding of CHIKV to the cell surface receptors but prevents the internalization of virus particles into the cells. Subsequently, the cells were warmed to 37°C, and the virus-infected cells were processed for embedding and sectioning at appropriate times after warming for transmission electron microscopy. At 5 mins upon warming to 37°C, CHIKV particles (Figure 2b, arrow) were observed to attach on the outer surface of the plasma membrane of C6/36 cells and CHIKV particles (Figure 2b, arrow) were also noted within invaginations of the plasma membrane. These invaginations resembled those of clathrin-coated pits (Figure 2b, arrowheads). Similarly, attachment and localization of CHIKV particles to clathrin molecules were revealed by double-labeled immunofluorescence staining of the cellular clathrin and CHIKV particles by specific antibodies (Figure 2c and Figure 2d).

**Figure 3:**
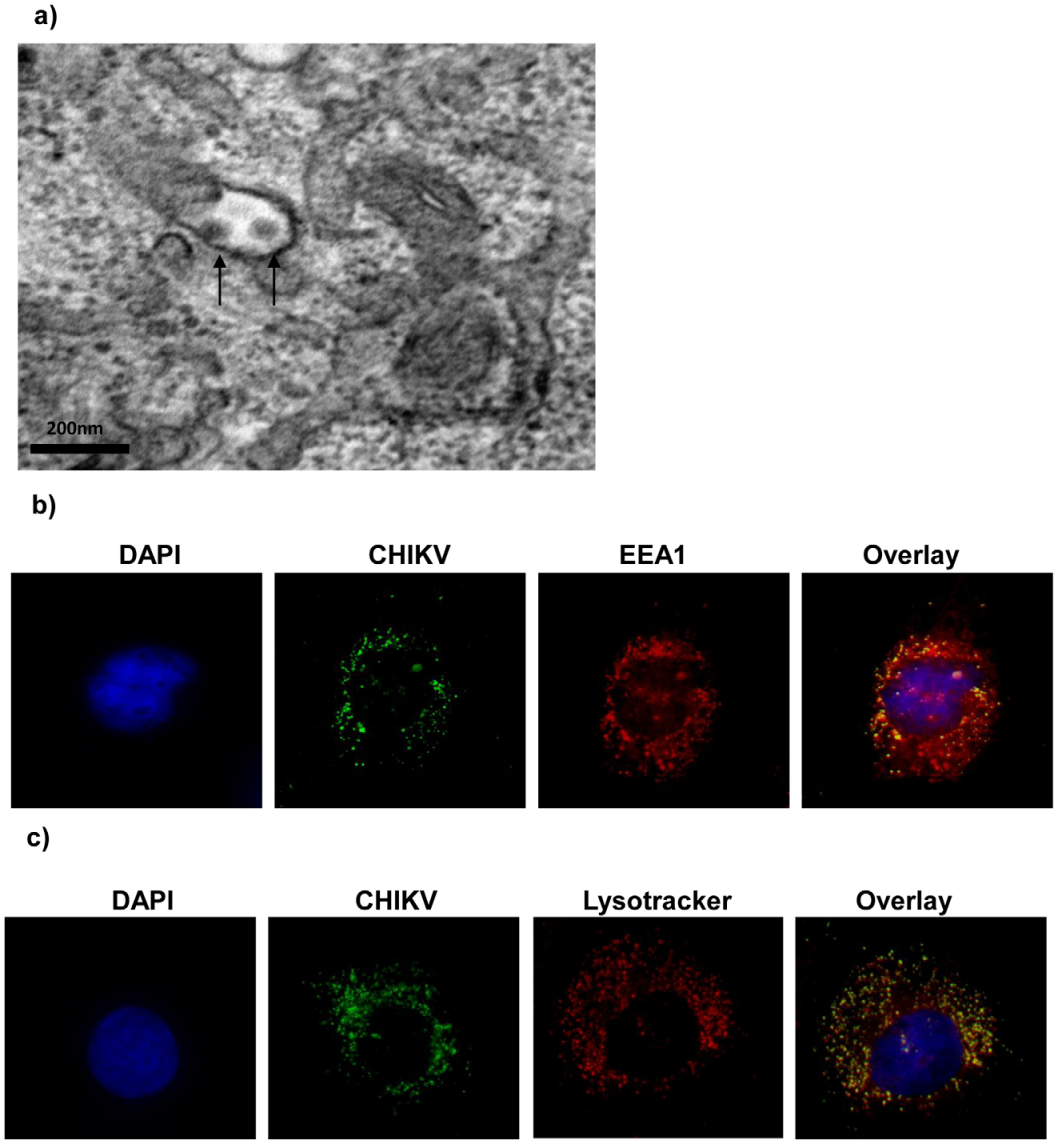
Colocalization of CHIKV with early and late endocytic vesicular markers within C6/36 cells. (a) TEM analyses reveal that CHIKV virus particles are contained within the endocytic vesicle (black arrows). Scale bar represents 200 nm. (b) Anti-EEA1 antibody (red) is used to stain the early endosomes at 10 mins p.i. Most of the virus-containing endosomes were distributed closer to the cell periphery. (c) Lysotracker (red) is used to stain late endosomes and lysosomes at 15 mins p.i. CHIKV particles are found mainly in the vesicles (green), suggesting that endocytosed CHIKV particles have been trafficked to the late endosomes and lysosomes.

After 10 mins at 37°C, most of the virus particles were observed within endocytic vesicles. CHIKV virus particles were contained within each of these vesicles (Figure 3a) as revealed at the ultrastructural level by transmission electron microscopy. These virus-containing vesicles were predominantly localized to the perinuclear region in close association with the endoplasmic reticulum (ER). To further characterize the origin of the cellular endocytic vesicles that were involved in the endocytic trafficking process of CHIKV, double-labeled immunofluorescence microscopy assays were performed. Antibodies specific for early endosomes (EEA1) and late endosomes and lysosomes (Lysotracker) were used. At 10 mins after cells were warmed to 37°C, a double-labeled immunofluorescence assay with anti-CHIKV envelope protein and anti-EEA1 antibodies showed colocalization mainly at the cell periphery region, suggesting that the virus particles were trafficked to the endosomes after endocytosis (Figure 3b). By 15 mins after incubation at 37°C, CHIKV particles were found mainly in vesicles (Figure 3c) that were stained with Lysotracker (Molecular Probes), thus indicating the trafficking of the endocytosed CHIKV particles to the late endosomes and lysosomes by this time point. The fluorescent staining was more intense at the perinuclear region. A unique accumulation of a large number of virus-containing late endosomes and lysosomes were observed at the perinuclear region by 15 mins (Figure 3c), and these structures remained predominant until 35 mins p.i. (data not shown).

### Drug Inhibitory Assays Confirm CHIKV Infectious Entry via Clathrin-Mediated Endocytic Pathway

The results presented above suggested the involvement of a clathrin-mediated endocytic pathway in CHIKV entry into C6/36 cells. In order to further characterize the pathway by which CHIKV enters C6/36 cells, studies of various drugs inhibiting endocytosis and related processes were performed in a dose-dependent manner. C6/36 cells were pretreated with drugs that selectively inhibit receptor-mediated endocytosis [monodansylcadverine [24]], clathrin-dependent endocytosis [chlorpromazine [23] and dynasore [25]] and caveolae-dependent endocytosis [filipin and nystatin [26]]. Involvement of inhibitors associated with other entry pathways such as macropinocytosis [EIPA [27], [28]] and cholesterol-dependent endocytosis [methyl-β-cyclodextrin [26]] was also evaluated. Furthermore, inhibitors targeting actin polymerization [cytochalasin B [23] and cytochalasin D [31]], microtubule polymerization [colchicine [31] and nocodazole [32]] were used to investigate the role of cytoskeleton during CHIKV entry. Treatment of inhibitors associated with the acidification of endosomes [concanamycin A and bafilomycin A [4], [29], [30] as well as the calcium channel flux [(nifedipine [33]] were also performed (Table S1). Minimal cellular cytotoxicity was observed in drug-treated cells throughout the spectra of concentrations used in these experiments.

**Figure 4:**
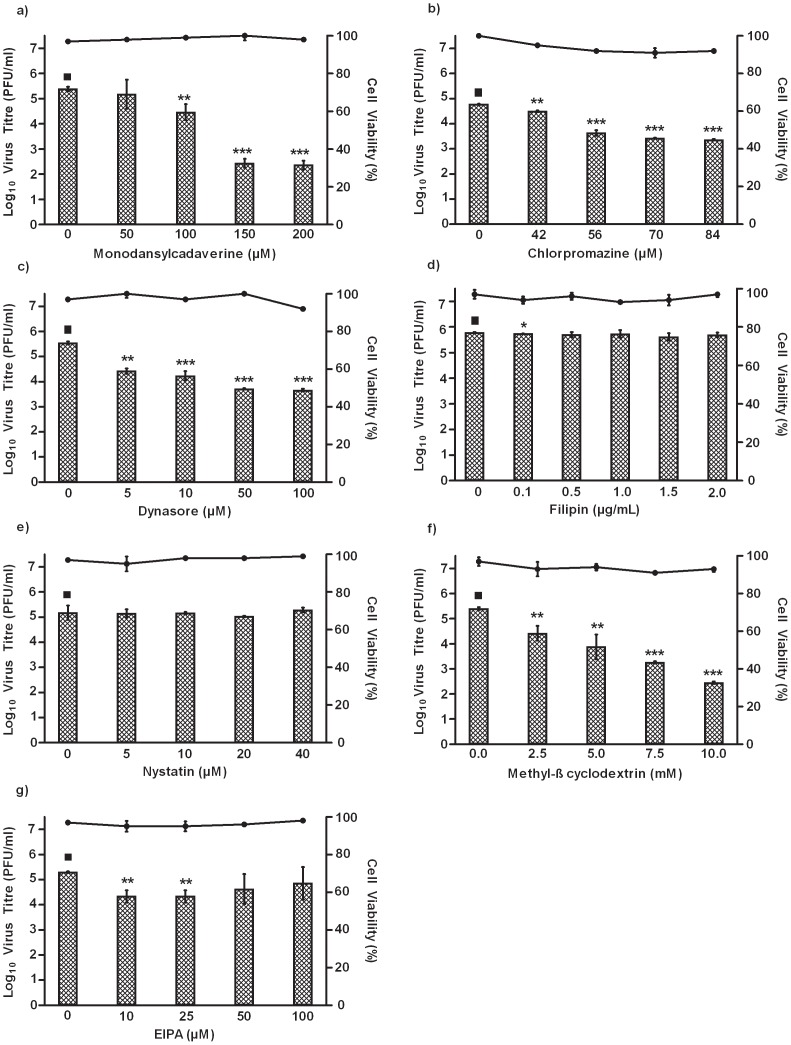
Effects of clathrin-mediated endocytic inhibitors on the entry of CHIKV into C6/36 cells. C6/36 cells were pre-treated with different inhibitors for 3 hours before CHIKV infection. Supernatants were harvested 24 hours p.i for viral plaque assays. The log virus titre is plotted against the concentrations of drug used. Dose-dependent inhibition of CHIKV entry into (a) monodansylcadaverine-, (b) chlorpromazine- and (c) dynasore-treated cells is observed. In contrast, minimal inhibition of CHIKV infectious entry into (d) filipin- and (e) nystatin-treated cells is noted. Cholesterol-dependent endocytosis of CHIKV into C6/36 cells is further analysed. Dose-dependent inhibition of CHIKV infection is observed with (f) methyl-β cyclodextrin treatment of C6/36 cells. Furthermore, minimal inhibition on the infectious entry of CHIKV into (g) EIPA-treated C6/36 cells is observed. Cell viability upon drug treatments is represented by the line graphs. The asterisk indicates *p values<0.05, **p values of <0.01 and ***p values<0.0001 by Student's *t* test using GraphPad Prism version 5.00 for Windows, GraphPad Software. Asterisks indicate statistically significant results relative to control group (▪).

Viral entry occurs via several endocytic pathways, with the most common being clathrin- and caveolae-mediated endocytosis [34], [35]. Drug treatment assays were carried out to determine whether CHIKV enters C6/36 cells via receptor-mediated endocytosis, and more specifically clathrin- or caveolae-mediated endocytosis. Upon treatment of monodansylcadverine, a well-known pharmacological drug inhibitor that targets receptor-mediated endocytosis [36], dose-dependent inhibition of CHIKV infection was observed, with a 2-log reduction at 150 µM (Figure 4a). Clathrin-mediated endocytic pathways can also be specifically inhibited by drugs such as chlorpromazine and dynasore. Chlorpromazine is a cationic, amphiphilic molecule that disrupts the assembly of clathrin lattices at the cell surface and endosomes [23], [26], whereas dynasore acts as a potent inhibitor of endocytic pathways by disrupting dynamin, thus preventing clathrin coated vesicles formation, [25]. Data revealed dose-dependent inhibition of CHIKV infection, upon treatment with chlorpromazine (Figure 4b) and dynasore (Figure 4c), showing 2-log reductions at 70 µM and 10 µM respectively. This suggests that CHIKV entry into C6/36 cells occurs via clathrin-mediated endocytosis. To eliminate the involvement of other entry pathways during CHIKV infection, drugs known to inhibit caveolae-mediated endocytosis and macropinocytosis were also evaluated. Caveolae-mediated drug inhibitors, filipin and nystatin inhibit virus entry by disrupting the caveolae, thus preventing caveolae formation [26]. Treatment with filipin (Figure. 4d) and nystatin (Figure 4e) did not exhibit inhibitory effects on CHIKV infection at any of the drug concentrations used. These results suggest minimal involvement of caveolae-mediated endocytosis upon CHIKV infection in C6/36 cells.

Early studies on *alphaviruses* have shown that lipid rafts are crucial players during virus entry, as cholesterol is needed to allow fusion of viruses with the endosomal membrane of host cells [37]. To evaluate the role of membranous cholesterol, treatment with methyl-β-cyclodextrin, a drug inhibitor targeting lipid raft synthesis via the removal of cholesterol by disrupting detergent-insoluble membrane micro-domains (DIMs) was evaluated in CHIKV infection [38], [39]. Results displayed dose-dependent inhibition, showing 2-log reductions at 2.5 mM (Figure 4f) suggesting that CHIKV entry is dependent on lipid raft synthesis targeting on membranous cholesterol. In a previous study, EIPA, an inhibitor of macropinocytosis, successfully inhibited rhinovirus 2 and Coxsackie B3 virus entry into HeLa cells [40]. However, in this study, at low concentrations of 10 and 25 µM, EIPA only displayed minimal inhibitory effects on the entry pathway of CHIKV infection. Instead, CHIKV infection was observed to be enhanced (Figure 4g). Possible reasons could include the activation of reflex mechanisms in cells, thus causing an increase of endocytic uptake through other pathways.

**Figure 5:**
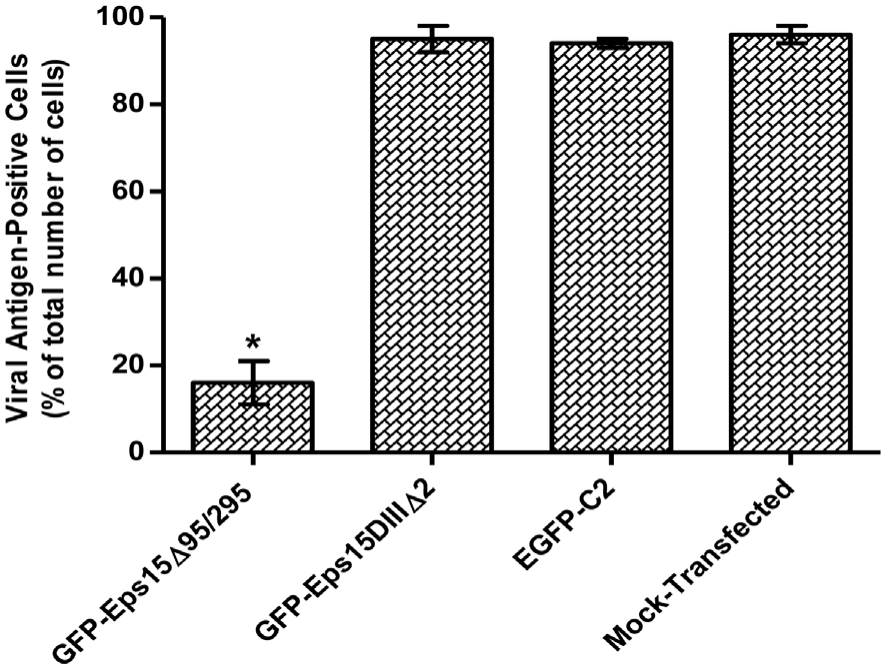
Dominant negative inhibitor of Eps15 inhibits infectious entry of CHIKV into C6/36 cells. The infectious entry of CHIKV into C6/36 cells is significantly inhibited when cells are transfected with GFP-Eps15Δ95/295 dominant negative plasmid construct whereas GFP-Eps15DIIIΔ2 and EGFP-C2 constructs serve as negative controls and have no effect on CHIKV entry into cells. The histogram represents the inhibition of virus entry as determined by the number of viral antigen positive cells in relation to the total cell population. The plots shown are representative of three independent experiments. The asterisk indicates *p* values of ≤0.05 by Student's *t* test.

The employment of dominant-negative mutants of Eps15 can be much more specific in targeting the arrestment of clathrin-coated pit formation [41]. GFP-tagged dominant negative mutant of Eps15, (GFP-EΔ95/295), GFP-tagged negative control constructs (GFP-D3Δ2) and internal GFP control were transiently transfected into C6/36 cells [42]. Transfection efficiencies for all constructs were observed to be more than 80% by fluorescence microscopy. Transfected cells were then assayed for their capability to internalize Texas Red- (TR-) conjugated transferrin, a specific marker for clathrin-dependent endocytosis. Indeed, at 48 hours post-transfection, maximal expression of the transfected gene can be observed and the internalization of TR-transferrin was impaired in cells transfected with GFP-EΔ95/295. In contrast, the uptake of TR-transferrin was not affected in cells expressing GFP-D3Δ2 or GFP (data not shown). The dominant negative mutant GFP-EΔ95/295 drastically inhibited CHIKV infection by more than 80% but neither of the control constructs exerted any inhibitory effects on CHIKV infection in C6/36 cells (Figure 5).

### Low Endosomal pH Involvement in CHIKV Entry

**Figure 6:**
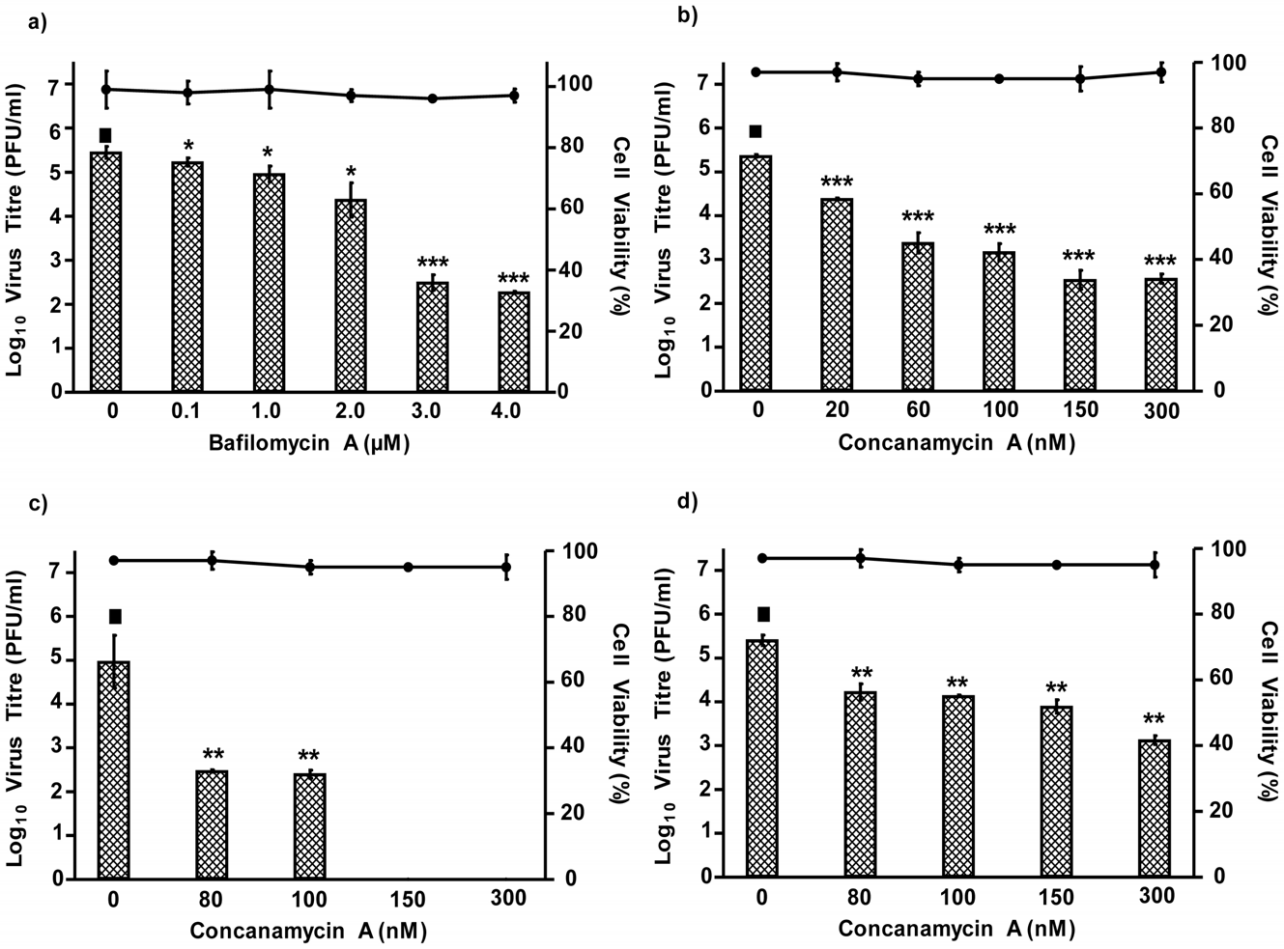
Effects of low endosomal pH inhibitors on the entry of CHIKV into C6/36 cells. C6/36 cells were pre-treated with different drug inhibitors for 3 hours before CHIKV infection. Supernatants were harvested 24 hours p.i for viral plaque assays. Low endosomal pH inhibitors show dose-dependent inhibition of CHIKV entry into (a) bafilomycin A-, (b) concanamycin A-treated cells, infected with CHIKV Singapore/07/2008 strain, (c) concanamycin A-treated infected with CHIKV SGEHICHD122508 strain and (d) concanamycin A-treated cells infected with CHIKV SGEHIDSD67Y2008 strain. The log virus titre is plotted against the concentrations of drug used. Cell viability upon drug treatments is represented by the line graphs. The asterisk indicates *p values<0.05, **p values of <0.01 and ***p values<0.0001 by Student's *t* test. Asterisks indicate statistically significant results relative to control group (▪).

Most enveloped viruses require low-endosomal pH to enter host cells via endocytosis, which is maintained by vacuolar proton-ATPases, to trigger fusion of the viral envelope with the endosomal membrane and release the nucleocapsid into the cytosol [31], [42], [43]. Drug treatment assays were performed to examine the low pH-dependence of CHIKV entry using the vacuolar proton-ATPase inhibitors, namely bafilomycin A1 - which inhibits endosomal and lysosomal acidification [29], [30] - and concanamycin A - which inhibits acidification of organelles [44] As shown in Figure 6, bafilomycin A1 and concanamycin A displayed dose-dependent inhibitory levels with at least 2-log reductions at 3 µM (Figure 6a) and 60 nM (Figure 6b) respectively. These results strongly suggest that CHIKV entry process is dependent on low endosomal pH.

In addition, recent studies reported that more sensitive inhibition of E1-226V mutated CHIKV LR-OPY1 strain upon endosomal pH acidification with bafilomycin A1 and chloroquine on *Ae. albopictus* cells were observed as opposed to CHIKV 37997 African reference strain [45]. Therefore, in our studies, C6/36 cells treated with concanamycin A were tested against local isolates of CHIKV, namely the SGEHIDSD67Y2008 strain, which is similar to the prototypic CHIKV 37997 African reference strain, and the SGEHICHD122508 strain, which closely resembles the E1-226V mutated CHIKV LR-OPY1 strain. Results displayed complete inhibition at 150 nM for the CHIKV SGEHICHD122508 strain (Figure 6c) when compared to the CHIKV SGEHIDSD67Y2008 strain (Figure 6d). These findings matched those observed by Gay *et al.* (2012), in which mutations in CHIKV strains result in more sensitive inhibitory levels upon endosomal pH acidification.

**Figure 7:**
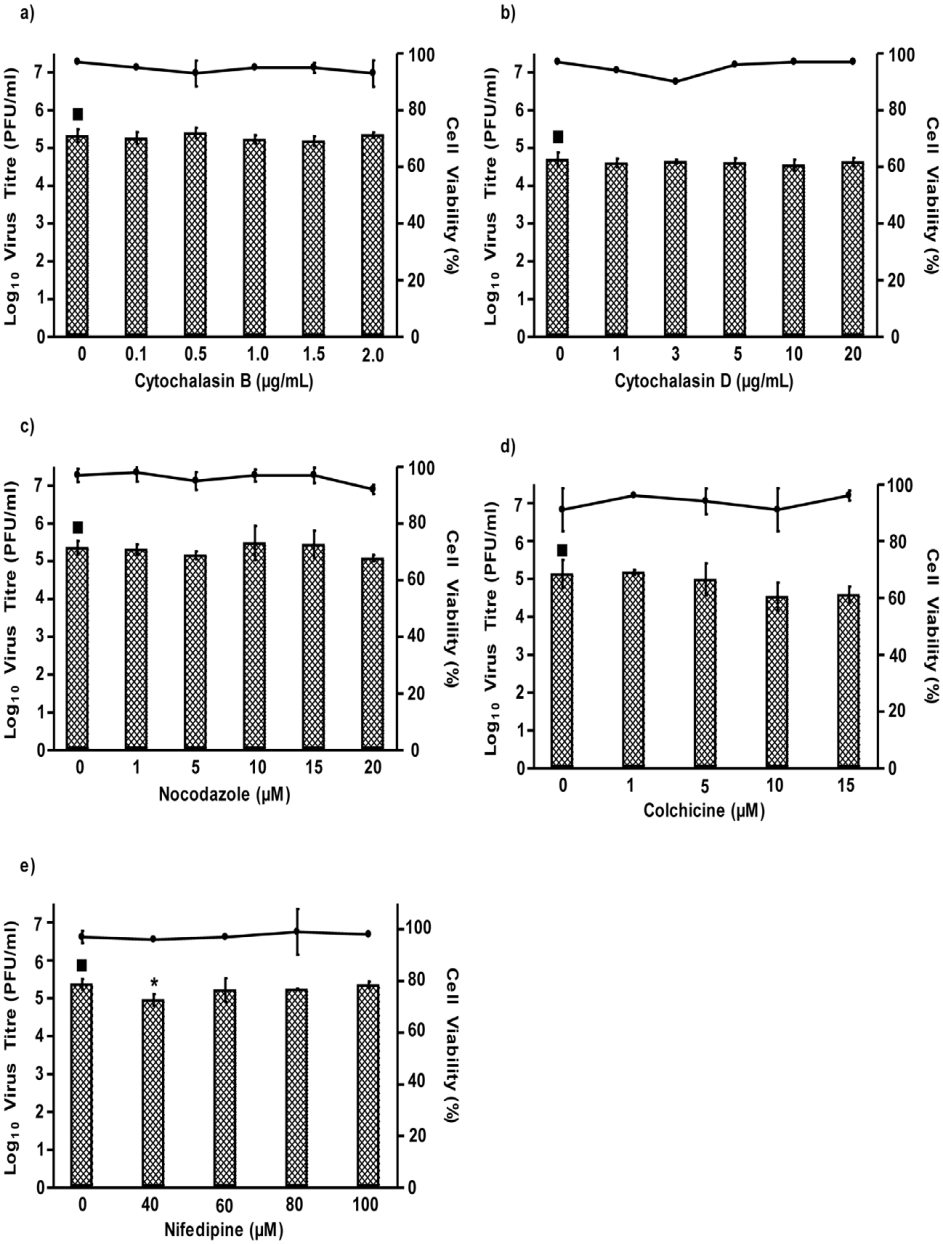
Effects of cytoskeleton disrupting drugs on the infectious entry of CHIKV into C6/36 cells. C6/36 cells were pre-treated with different drug inhibitors for 3 hours before CHIKV infection. Supernatants were harvested 24 hours p.i for viral plaque assays. Minimal involvement of actin was observed on CHIKV entry as revealed by the infectious virus titer of the (a) cytochalasin B- and (b) cytochalasin D-treated C6/36 cells as compared to mock-treated cells. Similarly, microtubules-disrupting drugs on CHIKV-infected C6/36 cells showed no inhibition on CHIKV entry into (c) nocodazole- and (d) colchicine-treated cells. (e) nifedipine has minimal effect on the infectious entry of CHIKV into C6/36 cells. The log virus titre is plotted against the concentrations of drug used. Cell viability upon drug treatments is represented by the line graphs. The asterisk indicates *p values<0.05 by Student's *t*. Asterisks indicate statistically significant results relative to control group (▪).

Involvement of the cellular cytoskeletal network on CHIKV entry was also investigated via treatment with cytoskeleton-disrupting drugs. Actin filaments have been shown to assist the initial uptake of ligands via clathrin-coated pits and the subsequent degradative pathway, whereas microtubules are known to be involved in maintaining endosomal traffic between peripheral early and late endosomes. Cytochalasin B and D are actin-disrupting drugs, which specifically target the actin cytoskeleton by preventing its polymerization into microfilaments and promoting microfilament disassembly [44]. Pretreatment of cells with cytochalasin B and D (Figures 7a and 7b respectively) failed to inhibit CHIKV infection. Similarly, treatment with nocodazole (Figure 7c) and colchicine (Figure 7d), inhibitors resulting in depolymerization of microtubules, showed no inhibition of CHIKV infection, thus indicating that CHIKV entry does not rely on microtubule polymerization [31]. These results suggest minimal involvement of the cytoskeletons in the entry process of CHIKV infection.

Previous studies on herpes simplex viruses have identified the importance of calcium (Ca^2+^) flux in virus entry for delivering virus capsids to the cytoplasm or nucleus [33]. Therefore, to determine whether Ca^2+^ flux is important in CHIKV infection, nifedipine, an inhibitor of dihydropyridine L-type voltage sensitive Ca^2+^ channel flux, was used. However, in this study, nifedipine treatment (Figure 7e) failed to inhibit CHIKV infection, thus indicating that Ca^2+^ flux is not required for CHIKV infection. From these drug treatment assays, it can thus be concluded that CHIKV entry into C6/36 cells occurs via clathrin-mediated endocytosis. Low endosomal pH is found to play a significant role in CHIKV entry, while the cytoskeleton and Ca^2+^ flux may not be vital for the endocytic process of CHIKV infection.

### Knockdown of Gene Expression of Targeted Cellular Genes

Data from the microarray analyses has revealed the differentiated regulation of genes associated with the clathrin-mediated endocytic pathway, and drug treatment assays have validated the involvement of the pathway in the infectious entry of CHIKV into mosquito cells. To investigate the functional roles of genes related to clathrin-mediated endocytosis, siRNAs targeting clathrin-heavy chain (CLTC), Rab proteins (RAB5 and RAB7) and vacuolar ATPases (vacuolar ATPase B) were utilized in further downstream studies. Dose-dependent siRNA-based knockdown of the selected targeted cellular genes was performed in varying siRNA concentrations (0.1, 1, 5, 10 nM) on C6/36 cells, prior to being subjected to CHIKV infection. Scrambled siRNAs were included as controls to ensure the specificity of the siRNAs used in this study. Minimal cellular cytotoxicity was observed in siRNA-treated cells throughout the spectra of concentrations used in these experiments (data not shown). RNA expression levels of the knocked-down genes were analyzed, with the non-infected samples being harvested at 48 hours post transfection. Significant reduction was observed in the levels of gene expression of CLTC, RAB5, RAB7 and vacuolar ATPase B relative to non-transfected cells (TC) (Figure S2a–S2d, solid bars). In contrast, data for scrambled siRNA gene expression showed similar levels of gene expression to TC samples (Figure S2a–S2d, striped bars). These results suggested that the siRNA knockdown of the targeted cellular genes is specific.

**Figure 8:**
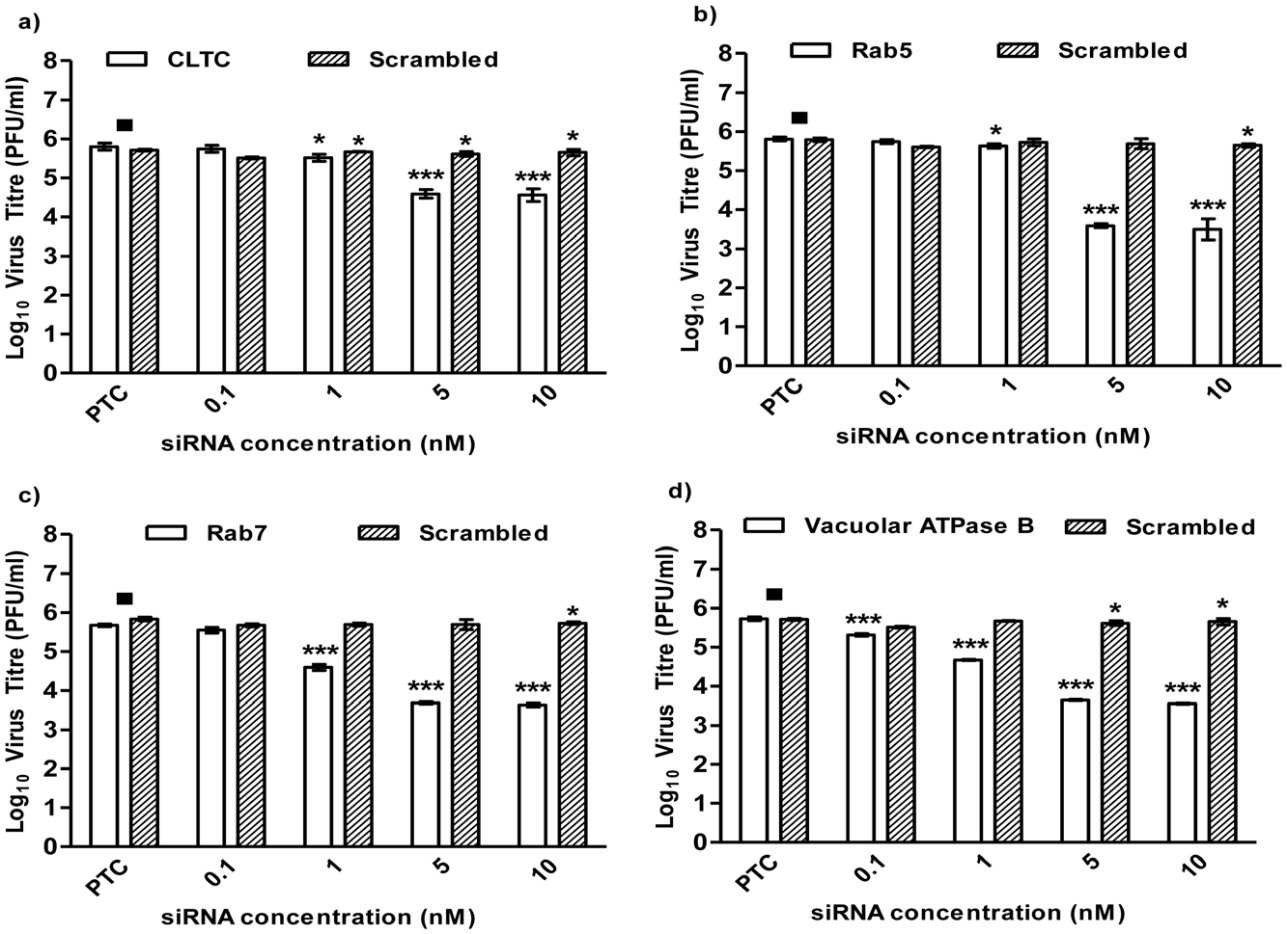
siRNA-based knockdown on cellular genes of CLTC, RAB5, RAB7 and vacuolar-ATPase B. Scrambled siRNAs (represented by striped bars) against (a) CLTC, (b) RAB5, (c) RAB7 and (d) vacuolar ATPase B were transfected into C6/36 cells across various concentrations (0–10 nM) and subjected to CHIKV infection. No virus inhibition is observed for all tested scrambled siRNAs when compared to non-transfected cells (TC). Gene-specific siRNAs (represented by solid bars) against (a) CLTC, (b) RAB5, (c) RAB7 and (d) vacuolar ATPase B are transfected into C6/36 cells at different concentrations (0–10 nM) and subjected to CHIKV infection. Significant dose-dependent inhibition of CHIKV infection are observed from 5 nM to 10 nM, with approximately 2-log reductions seen across all genes tested. The asterisk indicates *p values<0.05, **p values of <0.01 and ***p values<0.0001 by Student's *t* test. Asterisks indicate statistically significant results relative to control group (▪).

Effects of the scrambled siRNAs showed minimal inhibition of CHIKV infection relative to CHIKV-infected non-transfected cells (PTC) (Figure 8a–8d, striped bars). However, cells with specific siRNA knockdown of CLTC gene showed dose-dependent reduction in the infectious viral titre of CHIKV, with a 1-log reduction at 5 nM, relative to the PTC samples (Figure 8a, solid bars). siRNAs targeting the endosomal trafficking pathway (RAB5 and RAB7), which are involved in viral entry via the trafficking of the early and late endosomes, prevented CHIKV infection in a dose-dependent manner, showing a 3-log reduction in infectious virus titre at 5 nM RAB5 siRNA (Figure 8b, solid bars). A 1-log reduction in CHIKV titre at 1 nM RAB7 siRNA (Figure 8c, solid bars) further accounts for the trafficking of internalized CHIKV particles from early endosomes to the late endosomes. In addition, silencing of vacuolar ATPase B, involved in endosomal acidification, also led to a decrease in CHIKV infection in a dose-dependent manner, with a 2-log reduction at 5nM (Figure 8d, solid bars). These results further confirmed our earlier findings that CHIKV entry into *Ae. albopictus* (C6/36) cells occurs via clathrin-mediated endocytosis and is dependent on low pH endosomal acidification.

## Discussion

Interest on deciphering virus entry into host cells has been steadily gaining momentum over recent years, in the hope to establish potentially powerful anti-viral strategies against these medically important human pathogens. Studies have shown that numerous viruses enter via receptor-mediated and/or clathrin-mediated pathways [34], [35]. The entry process of many enveloped viruses typically begins with the fusion of viral envelope glycoproteins at the plasma membrane allowing internalization of viral nucleocapsids at neutral pH. Virus entry can also occur via endocytosis prior to fusion with the endocytic membrane, whereby hydrophobic virus fusion proteins undergo conformational changes upon exposure to acidic pH resulting in the release of viral nucleocapsids into the cytoplasm. Receptor-mediated endocytosis forms the predominant mode of entry, often mediated by the formation of clathrin-coated pits, prior to subsequent transport of viruses to early endosomes, where the low pH environment triggers fusion [46]. Meanwhile, clathrin-mediated endocytosis primarily entails the binding of extracellular cargo molecules to specific cell-surface receptors. These receptors, along with other membrane proteins entering via endocytosis, are transported by the intracellular adaptor proteins to endocytic sites. Together with clathrin, the adaptor protein forms an enclosed coat at the plasma membrane. The coated membrane then bends to form invaginations resembling clathrin-coated pits that pinch off to form cargo-filled vesicles [47].

Nevertheless, analyses of these entry modes have been predominantly demonstrated in mammalian cells. Indeed, the involvement of endocytic pathways in the entry of alphaviruses has been extensively studies, with SFV and SINV found to penetrate target cells through clathrin-dependent endocytosis [3], [15], [48], [49]. Few studies have however been documented on endocytic entry pathways of arboviruses into mosquito cells. This study shows, for the first time, CHIKV infectious entry into *Ae.* mosquitoes cells via clathrin-mediated endocytosis. Although a recent study has shown CHIKV entry in mammalian cells via clathrin-independent endocytosis [3], [4], [7], earlier findings indicated the dependence of CHIKV infectious entry in mammalian cells on clathrin [3], [4], [7]. This work thus indicates that the infection mechanism in mosquitoes and mammals may have indeed occurred through a common conserved endocytic pathway.

A variety of experimental approaches was used in this novel study including microarray gene profiling, bioimaging studies (transmission electron microscopy, double-labeled immunofluorescence microscopy), pharmacological inhibitors, overexpression of dominant-negative mutant of Eps15 and siRNA-based knockdown of genes involved in the endocytic pathway.

A customized gene expression microarray was first conducted to identify host genes necessary for the infectious entry of CHIKV into mosquito cells. Several genes that were differentially regulated during CHIKV infection have been known to be involved in clathrin-mediated endocytosis (Table 1), including EPN1, EPS15 and HIP1. EPN1 is an accessory protein that interacts with EPS15 - a clathrin-coat-associated protein that binds the α-adaptin subunit of the clathrin adaptor AP2 (AP2A1) [50] - and clathrin, as well as with other accessory proteins for the endocytosis of clathrin-coated vesicles. It facilitates the rearrangement of the clathrin lattice, resulting in the formation of clathrin-coated invaginations and fission [51]. HIP1 plays a role in clathrin-mediated endocytosis and trafficking by regulating clathrin assembly via binding to a highly conserved region of clathrin light chain [52]. The microarray analysis also revealed the involvement of kinase-targeting genes (MAP2K7, MAP4K4 and MAPK14) - associated with the signal transduction processes of viral entry [53] – during early CHIKV infection. In addition, ATP6V1B2 and ATP6V1F, components of V-ATPases, were also found to be differentially expressed during the initial phases of CHIKV infection. This suggests a significant role for V-ATPases, which have been identified in intracellular compartments such as clathrin-coated vesicles and endosomes and are therefore essential in clathrin-mediated endocytosis [54]. The upregulation of ARFRP1 suggests the importance of vesicle and endosomal transport in early CHIKV infection, while the downregulation of RAB34 - which is a member of the Rab family small GTP-ases that regulates vesicle budding, docking and fusion, and has been predominantly associated with membrane ruffles and macropinosomes and promotes macropinosome formation [55] – eliminates the possible engagement of micropinocytosis for CHIKV infectious entry into mosquito cells. Taken together, analysis of the microarray data suggests that CHIKV entry occurs via clathrin-mediated endocytosis.

Downstream assays were subsequently performed in order to validate the microarray findings. Transmission electron microscopy analyses showed the presence of CHIKV particles within invaginations of the plasma membrane, resembling those of clathrin-coated pits. Furthermore, characterization of the vesicles involved in the endocytic trafficking processes of CHIKV revealed the translocation of the virus particles to early endosomes and subsequently to late endosomes and lysosomes. To this end, double-labeled immunofluorescence assays were performed with the early endosomal marker, EEA1 and late endosomal and lysosomal marker, Lysotracker. Colocalization of virus particles were observed upon double-labeling with anti-CHIKV envelope protein and anti-EEA1 antibodies, thus indicating the trafficking of CHIKV particles to endosomes upon entry into mosquito cells. These endosomes were also observed to be closer to the cell periphery. Subsequent labeling with Lysotracker showed that endocytosed CHIKV particles were trafficked from early to late endosomes and lysosomes (Figure 3c).

Further analyses of CHIKV internalization into C6/36 cells was determined by treating cells with a set of pharmacological inhibitors targeting receptor-, clathrin-, caveolae- mediated endocytosis, cholesterol-dependent endocytosis and macropinocytosis. Significant results from treatment with monodansylcadverine, chlorpromazine and dynasore proved the involvement of receptor- and/or clathrin- mediated endocytosis (Figure 4a–c).

The importance of lipid rafts has been widely acknowledged, with studies showing that DIMS, found in the plasma membrane of cell surface, posses the ability to isolate cholesterol into the hydrophobic pocket, thus aiding in entry of viruses [38], including Simian virus 40 (SV40) [56]. Moreover, studies in RNA viruses, such as HIV-1, have determined virus entry into host cells via lipid rafts, and treatment with methyl-β cyclodextrin resulted in blockade of trans-epithelial transcytosis of HIV-1 and reduction of envelope fusion [57]–[60]. Similarly, we reported in this study that methyl-β cyclodextrin treatment showed inhibition of CHIKV entry via C6/36 cells, thus suggesting that the infectious entry process of CHIKV is dependent on lipid raft synthesis targeting membranous cholesterol. In contrast, treatment with inhibitors such as flilipin, nystatin and EIPA, had minimal effects on inhibiting CHIKV infection, thus eliminating the possibility of CHIKV entry via other pathways.

Earlier studies have shown that the mutant form of Eps15, EΔ95/295 which contains a 200-amino acid deletion, prevented the association with AP2, thus inhibiting the entry of VEEV via clathrin-mediated endocytic pathway [30]. We observed similar observations in this study, with the overexpression of EΔ95/295 found to reduce the infectious entry of CHIKV. It can therefore be concluded that CHIKV entry into C6/36 cells occurs via clathrin-mediated endocytosis.

Earlier studies have shown that E1 constitutes the fusion protein of the *alphaviruses* [61]–[63]. In the endosomal vesicles containing endocytosed CHIKV particles, the E1–E2 heterodimer undergoes a conformational change upon exposure to low pH. This causes rearrangement to a homotrimeric complex of E1 formation, leading to increased activity for membrane fusion [64], [65]. Membrane fusion processes occur rapidly via the insertion of hydrophobic fusion peptides to form pores in cellular and viral membranes [66], thus releasing the nucleocapsid into the cytoplasm of the cell even before the degradation of the lysosomes [67]. Requiring low pH endosomal exposure, *alphaviruses* exposed to lysotromphobic weak bases such as bafilomycin A1, chloroquine and concanamycin A, are unable to undergo membrane fusion due to neutralization of pH in the endosomes [67], [68]. For instance, infection of SFV on *Ae. albopictus* cells was inhibited upon treatment with inhibitors targeting low-endosomal acidification [66].

A recent study on E1-A226V mutated CHIKV LR-OPY1 strain showed that it is more sensitive to inhibition via endosomal pH acidification with bafilomycin A1 and chloroquine on *Ae. albopictus* cells as opposed to the CHIKV 37997 African reference strain [45]. These two strains possess 85% nucleotide sequence identity, differing only in the E1 protein at position 226 [9]. Furthermore, CHIKV infection of C6/36 cells was found to be sensitive to inhibitors of the v-ATPase and chloroquine, a weak base that accumulates in the acidic parts of the cell and inhibits the acidification of endocytic compartments [45]. Similarly, in our own study, we observed lower levels of inhibition for the CHIKV SGEHIDSD67Y2008 strain - which has features common to those of the CHIKV 37997 African reference strain - than those of the CHIKV SGEHICHD122508 strain, which resembles the E1-A226V mutated CHIKV LR-OPY1 strain. The results revealed that while both the CHIKV SGEHICHD122508 and SGEHIDSD67Y2008 strains require endosomal acidification for optimal infection of *Ae. albopictus* cells, the former is more sensitive to inhibition as compared to the latter. This could be due to the differential sensitivities of the CHIKV strains to lysomotropic agents and weak bases, as similarly reported in previous studies [3], [4].

Our findings in this study were also further evaluated via siRNA-based dosage dependence analyses of several cellular genes associated with clathrin-mediated endocytosis and endosomal acidification. siRNA targeted against CLTC showed significant inhibition of CHIKV infection, thus further strengthening our earlier findings, in which treatment with clathrin-mediated endocytic associated inhibitors showed similar dose-dependent inhibitory trends of CHIKV infection. Similarly, silencing of vacuolar ATPase B also led to a decrease in CHIKV infection, strongly demonstrating that CHIKV entry requires low endosomal pH. Previous studies have shown that RAB5 and RAB7 proteins are usually associated with the translocation of viruses from the early to late endosomes [69]. In particular, mammalian cells infected with SFV were found to require the integrity of RAB5 proteins for productive infection [4], while RAB5 and RAB7 proteins were identified to play significant roles in the productive infection of vesicular stomatitis Indiana virus (VSV) and SFV in mosquito cells [69], [70]. Similarly, we observed significant inhibition of CHIKV infection upon siRNA-based knockdown of these genes, thus suggesting that CHIKV entry involves the translocation from early endosomes after clathrin-mediated endocytosis to late endosomes.

Based on our unprecedented findings in this novel study, it can thus be concluded that CHIKV infectious entry into *Ae. albopictus* cells occurs via clathrin-mediated endocytosis and is dependent on low endosomal pH acidification and the presence of membranous cholesterol. Elucidation of the infectious entry of CHIKV into mosquito C6/36 cells will contribute towards better understanding of CHIKV pathogenesis, thus enabling future development of antiviral strategies against the infectious entry process of CHIKV.

## Supporting Information

Figure S1 **Differentially expressed genes upon CHIKV infectious entry.** Clathrin-mediated endocytotic pathway: genes or related genes found to be differentially expressed during CHIKV infection are shaded in grey [adapted from IPA 9.0 (Ingenuity Systems, Inc.)]
(TIF)

Figure S2 **Gene expression of RNA levels on cellular genes of CLTC, RAB5, RAB7 and vacuolar-ATPase B.** Cells transfected with scrambled siRNAs (represented by striped bars) show high levels of gene expression compared to non-transfected cells (TC) across all genes tested. Cells transfected with targeted cellular siRNAs (represented by solid bars) against (a) CLTC, (b) RAB5, (c) RAB7 and (d) vacuolar ATPase B showed significant knockdown across all genes tested compared to TC. The *asterisk* indicates *p values<0.05, **p values of <0.01 and ***p values<0.0001 by Student's *t* test. Asterisks indicate statistically significant results relative to control group (▪).
(TIF)

Table S1 **Concentrations and functions of inhibitory drugs used in this study.**
(DOC)
