# Supplementary figures and images for: Mosquito Cellular Factors and Functions in Mediating the Infectious entry of Chikungunya Virus
Source: PLoS Negl Trop Dis. 2013 Feb 7;7(2):e2050. doi: 10.1371/journal.pntd.0002050 (PMC3567007; doi:10.1371/journal.pntd.0002050)

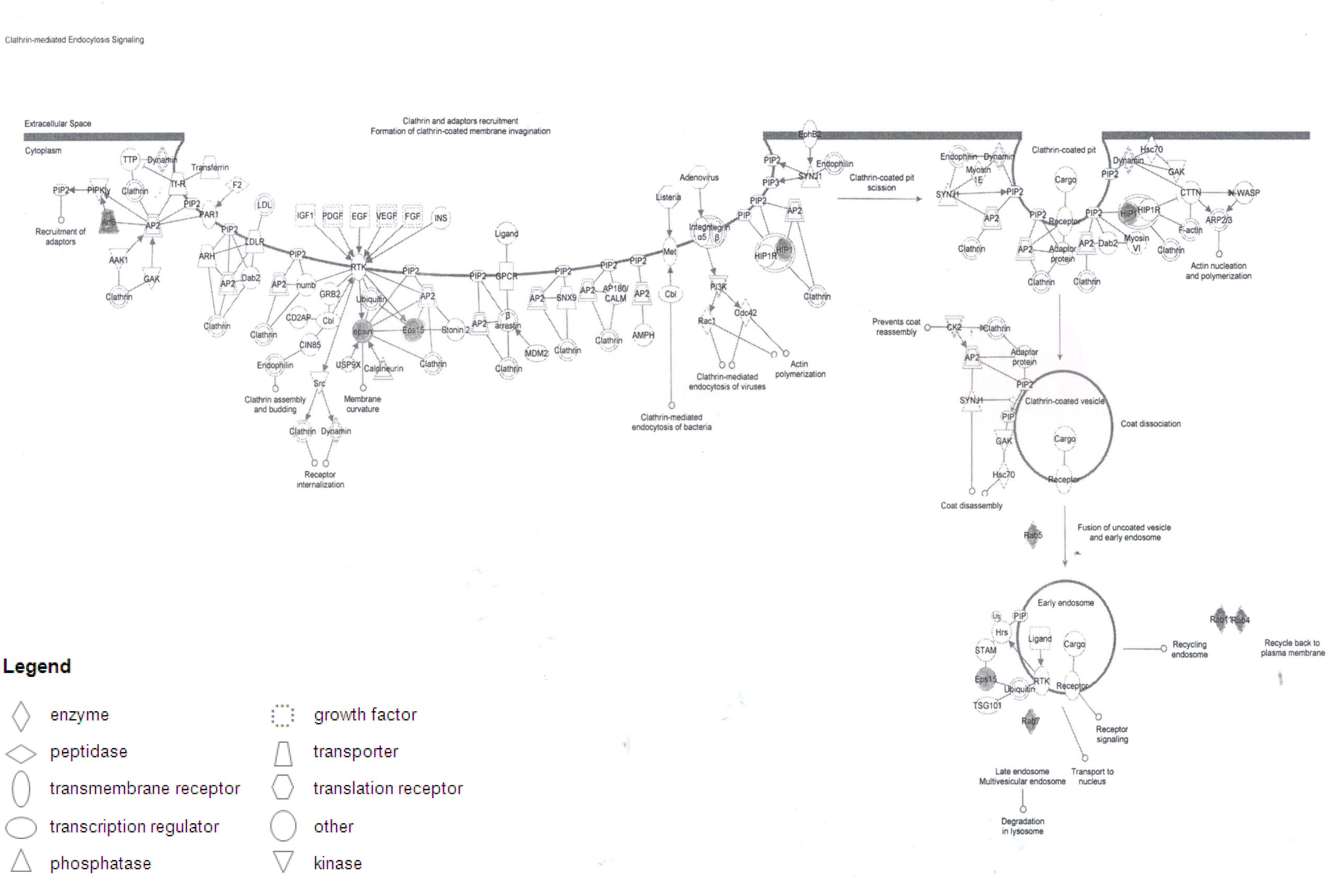

Supplement: Figure S1 — Differentially expressed genes upon CHIKV infectious entry. Clathrin-mediated endocytotic pathway: genes or related genes found to be differentially expressed during CHIKV infection are shaded in grey [adapted from IPA 9.0 (Ingenuity Systems, Inc.)] (TIF) [file pntd.0002050.s001.tif]

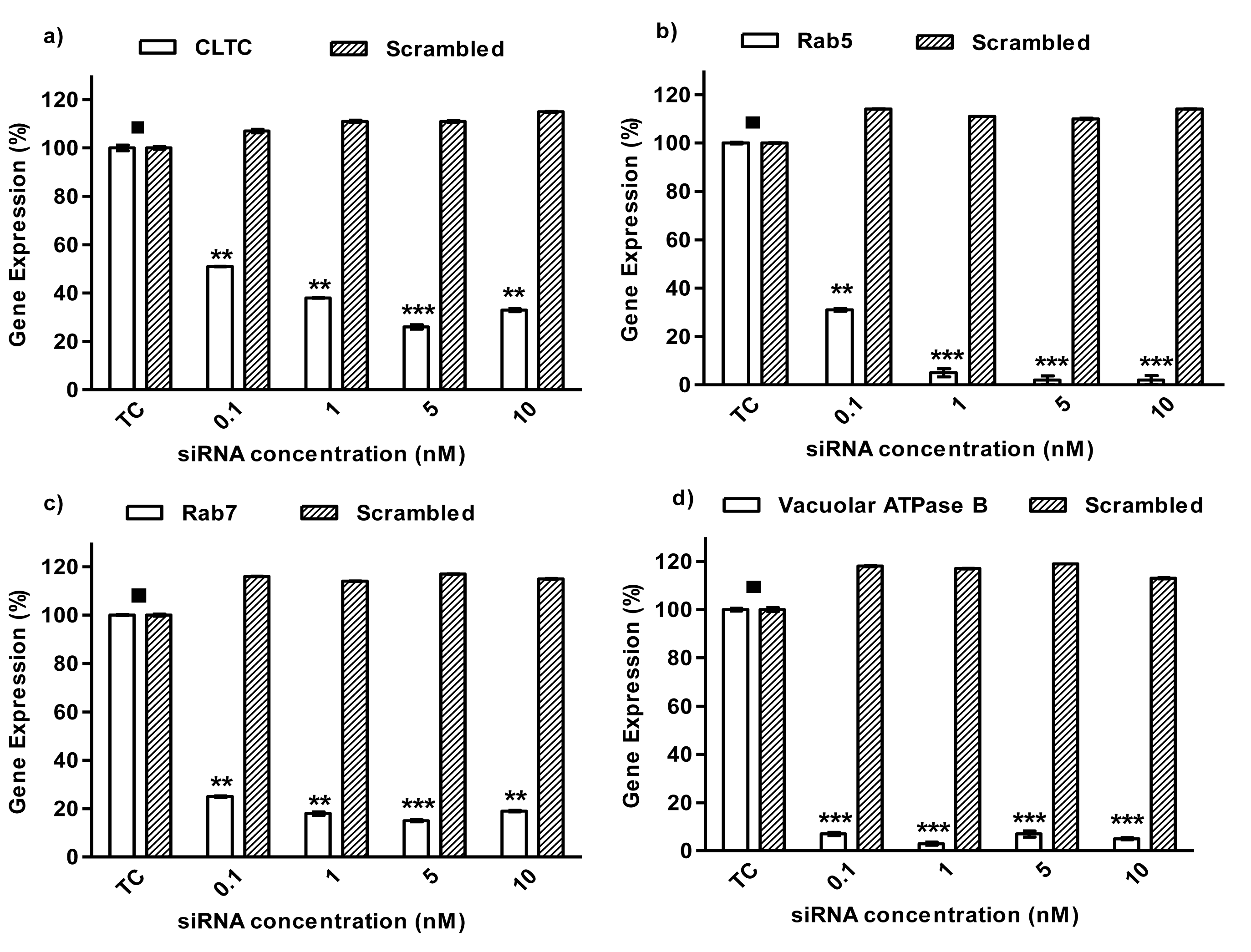

Supplement: Figure S2 — Gene expression of RNA levels on cellular genes of CLTC, RAB5, RAB7 and vacuolar-ATPase B. Cells transfected with scrambled siRNAs (represented by striped bars) show high levels of gene expression compared to non-transfected cells (TC) across all genes tested. Cells transfected with targeted cellular siRNAs (represented by solid bars) against (a) CLTC, (b) RAB5, (c) RAB7 and (d) vacuolar ATPase B showed significant knockdown across all genes tested compared to TC. The asterisk indicates *p values<0.05, **p values of <0.01 and ***p values<0.0001 by Student's t test. Asterisks indicate statistically significant results relative to control group (▪). (TIF) [file pntd.0002050.s002.tif]
