# Supplementary material for: Mosquito Cellular Factors and Functions in Mediating the Infectious entry of Chikungunya Virus
Source: PLoS Negl Trop Dis. 2013 Feb 7;7(2):e2050. doi: 10.1371/journal.pntd.0002050 (PMC3567007; doi:10.1371/journal.pntd.0002050)
Supplement: Table S1 — Concentrations and functions of inhibitory drugs used in this study. (DOC) [file pntd.0002050.s003.doc]

***SUPPORTING INFORMATION***

***Supporting Table S1. Concentrations and functions of inhibitory drugs used in this study:***

| **Drug** | **Concentrations used** | **Functions** |
| --- | --- | --- |
| Chlorpromazine | 42, 56, 70, 84 µM | Inhibitor of clathrin-dependent endocytosis (60) |
| Monodansylcadaverine | 50, 100, 150, 200 µM | Inhibitor receptor-mediated endocytosis (61) |
| Dynasore | 1, 5, 10, 50, 100 µM | Clathrin-coated vesicle formation in endocytosis (31) |
| Filipin | 0.5, 1.0, 1.5, 2.0 µg/mL | Inhibitor of caveolin-dependent endocytosis (42) |
| Nystatin | 5, 10, 20, 40 µM | Increases permeability of cell membrane of sensitive fungi by sterol binding (42) |
| Methyl-β-cyclodextrin | 2.5, 5, 7.5, 10 µM | Inhibitor of lipid raft synthesis and caveolin-dependent endocytosis (42) |
| 5-(*N*-Ethyl-*N*-isopropyl)-amiloride | 10, 25, 50, 100 µM | Selective blocker of Na+/H+ anti-port (35, 54) |
| Concanamycin A | 20, 40, 60, 80, 100, 150, 300 nM | Inhibitor of acidification of organelles and perforin-mediated cytotoxicity (4, 25) |
| Bafilomycin A | 0.1, 0.5, 1, 2, 3, 4 µM | Inhibitor of vacuolar H+ ATPase (4, 25) |
| Cytochalasin B | 0.1, 0.5, 1.0, 1.5, 2.0 µg/mL | Inhibitor of actin polymerization (60) |
| Cytochalasin D | 1, 3, 5, 10, 20 µg/mL | Inhibitor of actin polymerization (1) |
| Colchicine | 50, 100, 150, 200 µM | Inhibits microtubule polymerization by binding to tubulin (1) |
| Nocodazole | 1, 5, 10, 15, 20 µM | Inhibits microtubule polymerization by binding to tubulin (26) |
| Nifedipine | 40, 60, 80, 100 µM | Dihydropyridine L-type voltage sensitive calcium channel flux (10) |
